# Optimisation of Additives to Maximise Performance of Expandable Graphite-Based Intumescent-Flame-Retardant Polyurethane Composites

**DOI:** 10.3390/molecules28135100

**Published:** 2023-06-29

**Authors:** Imrana I. Kabir, Juan Carlos Baena, Wei Wang, Cheng Wang, Susan Oliver, Muhammad Tariq Nazir, Arslan Khalid, Yifeng Fu, Anthony Chun Yin Yuen, Guan Heng Yeoh

**Affiliations:** 1 School of Mechanical and Manufacturing Engineering, University of New South Wales, Sydney, NSW 2052, Australia; 2 Electrical and Biomedical Engineering, School of Engineering, RMIT University, Melbourne, VIC 3001, Australia; 3 School of Automotive and Traffic Engineering, Jiangsu University, Zhenjiang 212013, China; 4 Department of Building Environment and Energy Engineering, The Hong Kong Polytechnic University, Hung Hom, Kowloon, Hong Kong SAR, China; 5 Australian Nuclear Science and Technology Organisation (ANSTO), Locked Bag 2001, Kirrawee DC, NSW 2232, Australia

**Keywords:** polyurethane, composites, flame retardant, intumescent

## Abstract

The effect of varying the weight percentage composition (wt.%) of low-cost expandable graphite (EG), ammonium polyphosphate (APP), fibreglass (FG), and vermiculite (VMT) in polyurethane (PU) polymer was studied using a traditional intumescent flame retardant (IFR) system. The synergistic effect between EG, APP, FG, and VMT on the flame retardant properties of the PU composites was investigated using SEM, TGA, tensile strength tests, and cone calorimetry. The IFR that contained PU composites with 40 wt.% EG displayed superior flame retardant performance compared with the composites containing only 20 w.t.% or 10 w.t.% EG. The peak heat release rate, total smoke release, and carbon dioxide production from the 40 wt.% EG sample along with APP, FG, and VMT in the PU composite were 88%, 93%, and 92% less than the PU control sample, respectively. As a result, the synergistic effect was greatly influenced by the compactness of the united protective layer. The PU composite suppressed smoke emission and inhibited air penetrating the composite, thus reducing reactions with the gas volatiles of the material. SEM images and TGA results provided positive evidence for the combustion tests. Further, the mechanical properties of PU composites were also investigated. As expected, compared with control PU, the addition of flame-retardant additives decreased the tensile strength, but this was ameliorated with the addition of FG. These new PU composite materials provide a promising strategy for producing polymer composites with flame retardation and smoke suppression for construction materials.

## 1. Introduction

Accidental fires are a common occurrence in our living and working spaces, making fire safety of great importance when designing and constructing buildings. Indoor fires pose a significant health risk to occupants from exposure to volatile toxic compounds in smoke and hazardous particulates emitted from the fire [1,2,3,4]. This highlights a need to develop improved flame retardant (FR) materials that burn at a slower rate and expel less smoke and harmful gases. Improved FR materials allow occupants more time to evacuate buildings and reduce the risk of death. Therefore, the development of FR materials for construction purposes is critical for safety in future urban development [4,5].

Polyurethanes (PUs) are a large polymer class that are available in many forms, such as foams, composites, adhesives, and surface coatings. These capabilities allow them to be used in an array of industries, such as aerospace, construction, and textiles [1,2,3]. PUs’ properties include low thermal conductivity, resistance to abrasion, superb hardness, and low water absorption. Despite these favourable properties, PUs are highly flammable and emit large volumes of smoke when burnt [1,2,3,4,5,6]. With the rapid increase in PU usage owing to its wide range of applications, reducing its flammability is a priority. Further, studies show that the addition of various additives [7,8] and fillers [9,10] to the polymer matrix has a positive effect on the chemical and physical properties of the flame-retardant polymer composite, hence resulting in reduction and enhancing the flammability of the materials. For this to occur, the PU polymer needs to be modified with additives to improve its flame retardance [4,5,6].

Expandable graphite (EG), which functions as a smoke suppressor, is one of the most effective additive-type flame retardants. EG is more effective at smoke reduction than common FR additives, such as magnesium dihydroxide, aluminium trihydroxide, boric compounds, aluminium polyphosphate (APP), triphenyl phosphate, triethyl phosphate, layered silicate, melamine, and nanoparticles [11,12,13]. EG has a layered structure, with sulfuric acid (H_2_SO_4_) intercalated between the graphite flakes. When heated, the H_2_SO_4_ oxidises, expanding the carbon layer into a thick char that provides flame retardance [4]. The flame retardancy of the PU polymer matrix is further enhanced by combining EG with other flame retardants, such as APP, aluminium hypophosphite (AHP), and aluminium hydroxide (ATH) [14,15,16]. A PU composite sample containing 7.5 wt% EG with 7.5 wt% APP increased the limited oxygen index (LOI) value by 2.25% compared with the control PU [14]. Liu et al. [16] reported that the incorporation of dimethyl methyphosphonate (DMMP: 10 wt%, ATH: 5 wt%, APP: 15 wt%, EG: 20 wt%) in PU composites significantly improved the flame retardant properties when compared with the control PU composites. Xu et al.’s study showed that AHP (5 wt%) reinforced PU/EG (15 wt%) systems with high flame retardancy [17]. These studies [11,12,13,14,15,16,17] show that the incorporation of EG as a synergist is a useful way to improve EG’s flame retardant efficiency and reduce EG loadings in a PU polymer matrix.

Ammonium polyphosphate (APP) is another polymer additive that acts as an intumescent fire retardant, meaning that it forms an expanded char layer. This is due to phosphorus in APP altering the thermal degradation process, which leads to carbonisation and formation of the char layer through a condensable phase reaction [17,18]. Research demonstrated that, when combined with EG, APP reduced the heat release rate (HRR) and total heat release rate (THR), as well as reducing the smoke release rate (SRP) and total smoke production (TSP) [14,15,16]. The optimal ratio to maximise the synergistic effects of EG and APP was 2/3 [14,15,16]. A ratio of 10:5 was found to reduce smoke and flammability in wood flour and polypropylene composites by Song et al. [2]. A recent study by Xian-Yan Meng showed that, individually, APP and EG reduced the mechanical properties of the polymer, but, when combined, the properties increased, in turn reducing flammability [13]. Work by Chuigen Guo demonstrated the same effect, without a decrease in mechanical properties when the EG:APP ratio was 1:1 [11]. APP was found to control the rapid char layer expansion of EG, referred to as the “popcorn effect”, owing to the large chunks formed. While these formed a protective barrier, if the chunks were too large, they would break off, exposing the polymer below to the flame. When combined with APP, this “blow-off” effect was reduced, improving the flame resistance [12]. This research highlighted the improvements observed with the synergistic effects of multiple additives.

To maintain the mechanical strength of materials while increasing their fire retardant properties, fibreglass (FG) is a recommended additive. Fibreglass is widely used in polymers to increase strength, impact resistance, and recyclability [19]. However, owing to the structure of the glass fibres, they are susceptible to the “candlewick effect”, in which the flammable materials from the polymer are drawn through the glass fibres, accelerating the thermal degradation [20]. Therefore, more FR additives are required in polymers reinforced with FG. A study by Yun Liu et. al. [15] combined FR-reinforced polypropylene with a combination of intumescent flame retardants (a charring agent, APP, and organically modified montmorillonite). They demonstrated an increased flame retardance with only a small reduction in mechanical strength. A similar study used FG-reinforced polyester composites and EG, APP, and aluminium trihydroxide (ATH) as the intumescent fire retardants. A ratio of 50:50 wt.% for ATH:EG and 50:30:20 wt.% for ATH:APP:EG demonstrated the highest reduction in flammability without a loss of mechanical properties [21]. These studies demonstrated how synergising FR materials is optimal for increased flame retardance, while FG reinforcement maintains mechanical performance.

Vermiculite (VMT) is also a fire-retardant additive that can be combined with EG and APP in a PU polymer. VMT ((MgFe, Al)_3_(Al, Si)_4_O_10_(OH)_2_∙4H_2_O) is a hydrous silicate material with a triclinic structure and layers of minerals and water molecules. When heated above 200 °C, the bulk vermiculite exfoliates, releasing the water between the mineral layers, and this is its primary flame retardant mechanism. However, it is difficult to manufacture vermiculite films, especially for the thickness required (<40 μm) [22]. It was found that increasing the thickness of the films increased the fire retardance, as shown by a study Jun Young Cheong et al. [22]. In a study with VMT on fibreboard samples, the limiting oxygen index (LOI), and, therefore, flammability, was reduced with the addition of VMT; however, it also greatly reduced the mechanical strength [23]. By increasing the ratio of VMT in the particle board, the charring was over 10 times the volume of the untreated sample. These studies [22,23] showed the potential for VMT to be used for fire retardance; however, the drastic reduction in mechanical strength is still an issue to be overcome.

In this work, we have designed and characterised a new PU composite containing EG, APP, VMT, and FG for use in flame retardant applications. In particular, we have explored the effect of different additives on overall performance. This work will focus on the synergistic effects of this combination to improve the FR effects of the individual additives. This is a precursory step to produce cost-effective, low-toxicity, and environmentally benign PU composite membranes for construction materials to yield improved flame resistance performance.

## 2. Results and Discussion

### 2.1. Mechanical Properties: Tensile

**Figure 1:**
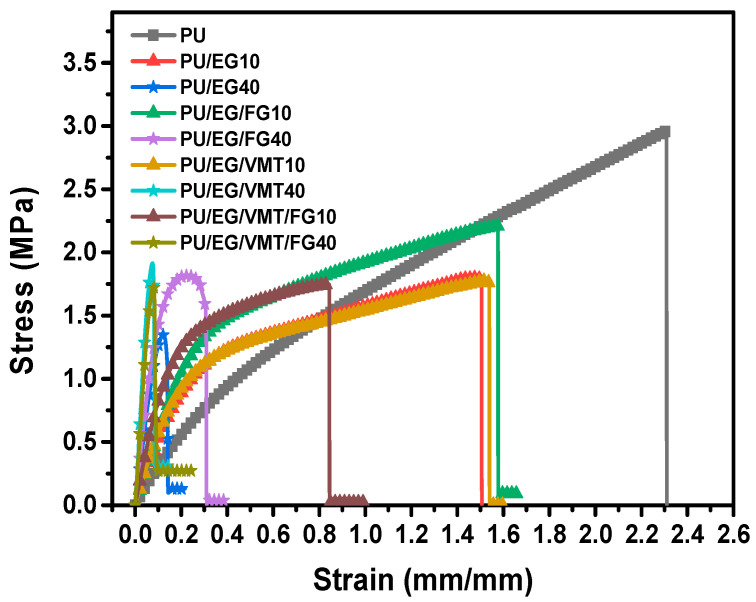
Tensile stress/strain curves for PU composites and control PU.

While reducing flammability is important in the material, its strength still must be retained for use in practical applications. As observed above in Figure 1 and Table 1, the control PU withstands the most stress before yielding at 2.86 MPa. This is followed by the PU/EG/FG10, PU/EG/VMT10, and PU/EG10 samples. The samples with higher proportions of additives had lower tensile strengths, which is a known drawback of intumescent fire retardants. It is important to note that the PU/EG/VMT/FG10 yielded at a significantly lower strain value than the other 10 wt.% samples, showing that the VMT has a negative impact on the tensile strength and flexibility. The PU/EG/FG40 sample also has the highest yield point of the 40 wt.% samples. This reflects the purpose of the fibreglass additive: to reinforce the polymer when the vermiculite and expandable graphite are added. The fibreglass reinforces the polymer and allows it to retain its structure when burnt rather than flowing when molten. This assists in ensuring that the sample and its charring layer can remain intact to prevent further burning.

### 2.2. SEM

**Figure 2:**
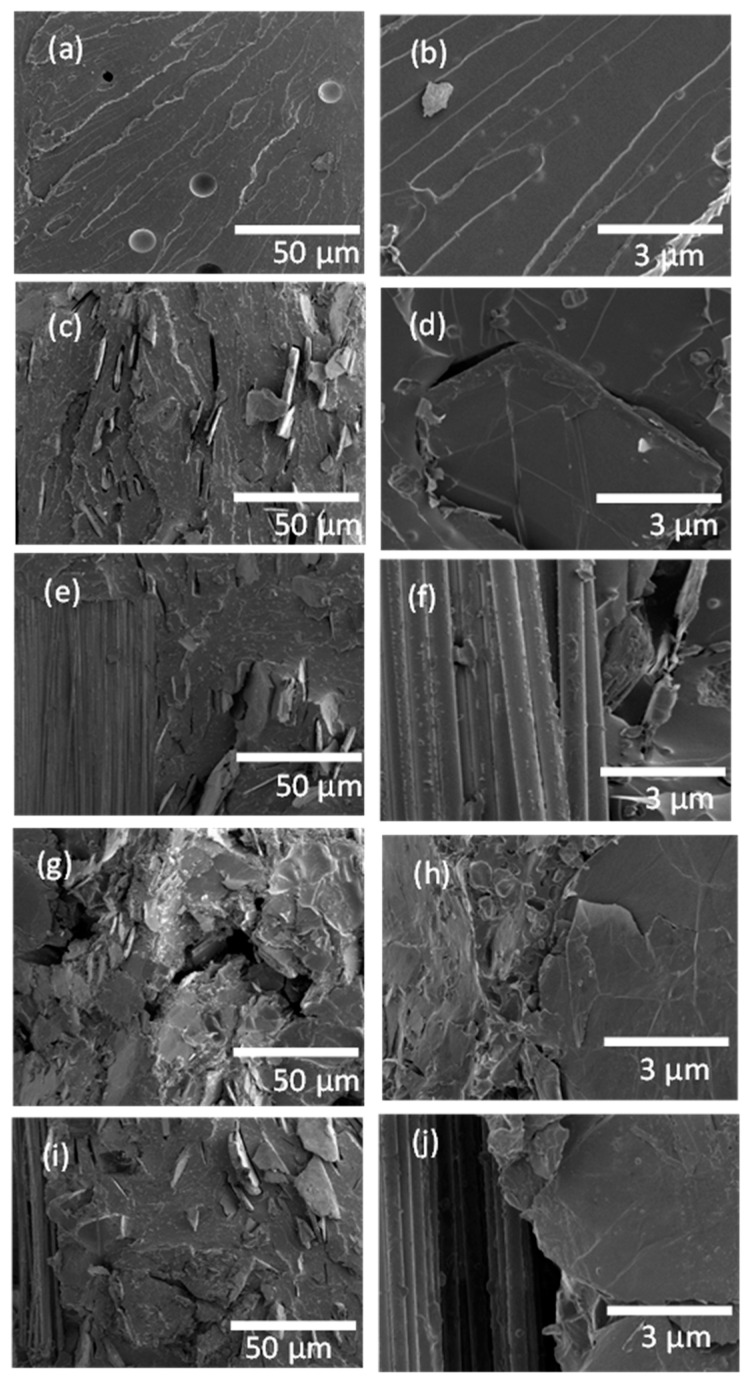
SEM micrograph surface at two different magnifications (50 k—left and 500 k—right) of (**a**,**b**) PU, (**c**,**d**) PU/EG10, (**e**,**f**) PU/EG/FG40, (**g**,**h**) PU/EG/VMT40, and (**i**,**j**) PU/EG/FG/VMT40.

To examine the surface morphology of the breakage point of the flame-retardant PU composites, a scanning electron microscope (SEM) was used at 50 k and 500 k magnification. From the SEM micrograph of the PU control sample (Figure 2a,b), it was observed that there were no noticeable matrix imperfections, with a smooth and even surface at the breakage point. This supports a reported study of pure PU material [1,2,3,4,5]. In Figure 2c,d, the EG is observed to damage the PU matrix compared with pure PU; PU/EG10 is larger in size and has a more irregular shape. This is owing to the EG increasing the viscosity during mixing, which hinders the dispersion of the graphite flakes. It was noted that, when adding APP (20 wt.%) to prepare PU/EG (In Figure 2c,d), it did not affect the homogeneity of the matrix. This is thought to be owing to the APP particles’ small size and how they are in the backbone of the polymeric matrix.

The compact glass fibre packs can be observed in (Figure 2e,f). The adhesion of the glass fibre to the matrix is hindered by the absence of characteristic functional groups and a plain surface, limiting the interaction surface. When the PU/EG/FG40 sample was broken, the glass fibre did not rupture. Cracks are more likely to occur on the phase border between the fibre and the matrix owing to the glass fibre’s high tensile strength and plain structure. This causes insufficient adhesion between the matrix and the glass fibre. A high roughness was observed at the PU/EG/VMT40 Figure 2g,h sample compared with the PU/EG10 and PU/EG/FG40 samples (Figure 2c–f). This high level of surface roughness can increase both the interaction surface and the adhesion of VMT to the PU matrix. The strong adhesion of VMT was also shown by the low number of small holes observed on the sample (Figure 2h) due to the VMT phases distributed through the polymer matrix.

As shown in Figure 2i,j, the surface morphology of the PU/EG/FG/VMT40 sample reveals FG and irregular graphite flakes. Less surface roughness was observed in the PU/EG/FG/VMT40 sample than the PU/EG/VMT40 despite VMT contributing to the effect in both. It is likely caused by the VMT improving the dispersion of the PU/EG/FG/VMT40 sample (Figure 2i,j) by reducing agglomeration and imperfections, as shown in Figure 2i,j.

### 2.3. Thermal Properties: TGA/DTG

**Figure 3:**
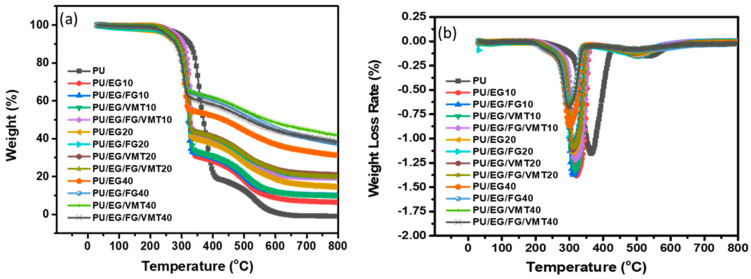
(**a**) TGA thermograms of PU composites and control PU under oxygen gas flow; (**b**) DTG thermograms of PU composites and control PU under oxygen gas flow.

Weight loss during TGA is indicative of material burning. A steep decrease can be observed at around 300 s after exposure in Figure 3a. The samples with the 40 wt.% EG were shown to reach a higher plateau of approximately 40%, with PU/EG40 being an exception and steadying at around 35%. The 20 wt.% and 10 wt.% EG samples reached as low as 10%, while the control PU sample burned almost completely, leaving virtually no material. The control PU initially began decreasing weight more gradually than all the other PU composite samples, as observed from the line deviating from the others in Figure 3a. However, this initial weight loss was greater than the other samples, falling to below 20% before flattening. The sudden loss of weight from the other samples may have been owing to the intumescent additives forming the barrier layer quickly, and some of the expanded material may have been combusted faster.

The weight loss rate in Figure 3b depicts a similar trend to that in Figure 3, with the combustion of the samples characterised by the steep increase in the weight loss rate followed by a plateau. Again, the samples with the 40 wt.% additives were observed to have a lower maximum rate. In addition, the curve for the PU sample is skewed to the right, showing a delayed thermal degradation that was less rapid but of longer duration.

### 2.4. Flammability Properties: Cone Calorimeter

**Figure 4:**
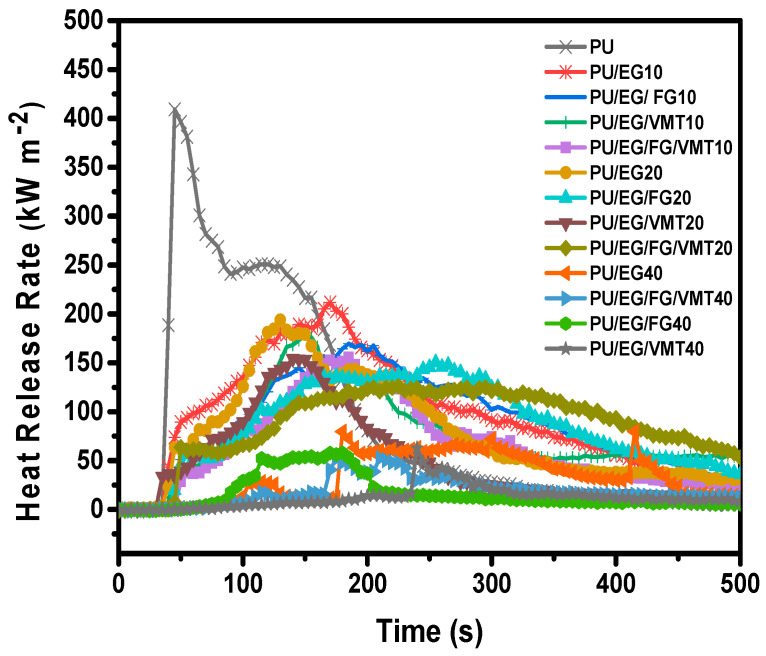
HRR as a function of the burning time for PU composite samples.

From Figure 4, the impact on the additives is clear from the sharp increase in the peak heat release rate (pHRR) of the control PU sample compared with the other samples. The PU control peaks at around 420 kWm^−2^ after 50 s of burning, indicative of very sudden burning after ignition. The samples of PU/EG10 and PU/EG20 reached peaks of 220 kWm^−2^ and 190 kWm^−2^ at 180 s and 130 s, respectively, showing a slightly slower and less intense thermal decomposition. When the additives had a 20 wt.% proportion, further reduction in HRR was observed, such as the PU/EG/FG20 and PU/EG/FG/VMT20 samples, which did not have a sudden peak. Furthermore, they reached values of 150 kWm^−2^ and 125 kWm^−2^ at 250 s, which showed that the additives decreased the flammability. This was through the EG and VMT forming a charring layer to prevent the flame from reaching the unburnt polymer matrix below. The slightly lower HRR value of the PU/EG/FG/VMT20 sample highlights the added impact of the exfoliation effect of the vermiculite. Both the PU/EG/FG40 and PU/EG/VMT40 had significantly lower maximum HRR values, with both peaking around 50 kWm^−2^ and remaining at 0 for the remainder of the experiment. It is important to note that the PU/EF/FG40 sample had a peak at 100 s before returning to 0 at 200 s, with PU/EG/VMT40 peaking at 150 s before returning to 0 at 300 s. This shows that the 40 wt.% samples burnt for a very short amount of time (with the exception of PU/EG40), and that the vermiculite was a more effective fire retardant than the fibreglass. The exception of PU/EG40 could have been caused by the char expanding too quickly and breaking off due to the high amount of EG. This is known as the “popcorn effect” [24,25,26] and is observed in other studies of EG [24,25,26].

**Figure 5:**
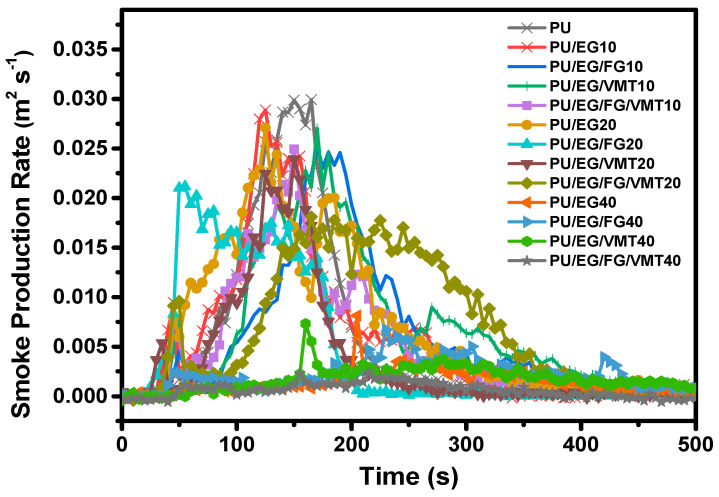
SPR as a function of the burning time for PU composite samples.

Smoke production rate (SPR) (Figure 5) is a critical measure for the lethality of fires. Consistent with HRR, the samples with the higher 40 wt.% of additive showed significantly lower smoke production, with the PU/EG/FG/VMT40 and PU/EG/VMT40 remaining at around 0 m^2^s^−1^ for the majority of the experiment. Research has shown that both EG and VMT can significantly reduce the smoke production of burning polymers [22,23]. The PU control showed the highest peak at 0.030 m^2^s^−1^ at around 150 s from ignition and again at 180 s. Most of the other samples of 10 wt.% and 20 wt.% peaked between 0.022 m^2^s^−1^ and 0.027 m^2^s^−1^ at 100–200 s before declining to 0 m^2^s^−1^ at around 250 s. Two notable exceptions were the PU/EG/FG20 and the PU/EG/FG/VMT20 samples. PU/EG/FG20 experienced an early peak of 0.033 m^2^s^−1^ at 50 s; however, Figure 5 did not indicate an early ignition. This shows that the sample emitted smoke before it began to release a significant amount of heat with the addition of FG. The PU/EF/FG/VMT20 showed a sustained peak of 0.017 m^2^s^−1^ from 150 to around 300 s, indicating a high release of smoke. This suggests that the fibreglass can increase smoke production when combined with other additives at 20 wt.% as this was not observed at 10 w.t.% or 40 w.t.%.

**Figure 6:**
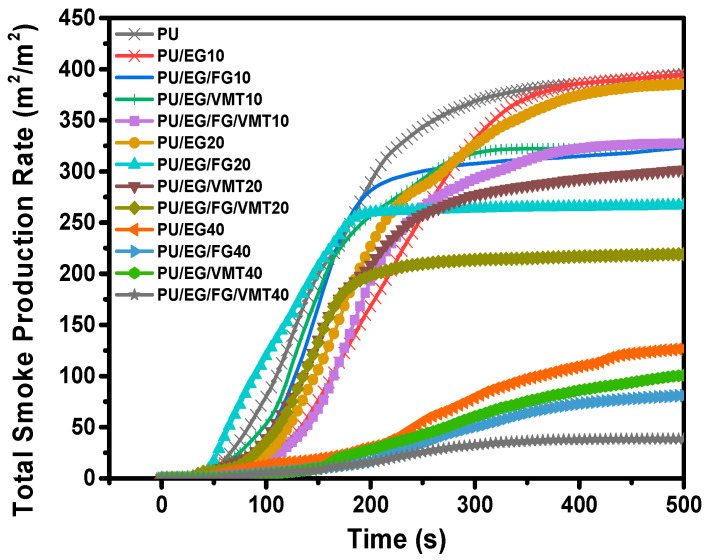
TSR as a function of the burning time for PU composite samples.

The total smoke production rate (TSR) in Figure 6 reflects what was observed in Figure 5, with the 40 wt.% samples producing a significantly lower amount of smoke compared with the samples with a smaller proportion of additives. This reiterates the smoke reduction effects of EG and VMT. The PU/EG/FG/VMT40 sample had the lowest value of 25 m^2^/m^2^. The control PU, PU/EG/10, and PU/EG20 samples reached a peak of 370 m^2^/m^2^, nearly 15 times the value of the PU/EG/FG/VMT40 smoke production rate. With the addition of FG and VMT at 10% wt., this value was observed to drop to around 330 m^2^/m^2^. This shows that fibreglass did reduce the total smoke production despite the early peak observed in the PU/FG/EG20 sample in Figure 5. Again, a clear decrease in smoke production (and, therefore, thermal degradation) is observed with an increase in additives.

**Figure 7:**
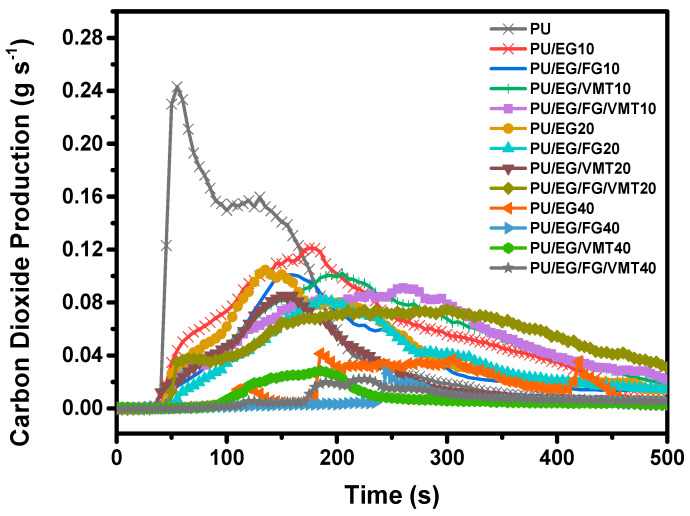
CO_2_P as a function of the burning time for PU composite samples.

The carbon dioxide production (CO_2_P) in Figure 7 strongly resembles the HRR in Figure 4 as the thermal degradation releases CO_2_. Again, the PU control sample is observed to have a sudden peak as it ignites the fastest after ignition. This peak is at 0.24 gs^−1^ at 50 s. The 40 wt.%. samples are shown to release very little CO_2_, with the most active sample, PU/EG40, releasing a maximum of 0.02 gs^−1^. This is due to the char layer forming and preventing the further burning of the polymer. As VMT releases water when heated, this further suppresses the burning and, therefore, CO_2_ release. The samples with 10–20 wt% additives showed steady production rates around 0.08 gs^−1^. This further shows that the increase in the proportion in flame-retardant additives reduces the flammability and, therefore, carbon dioxide production.

**Figure 8:**
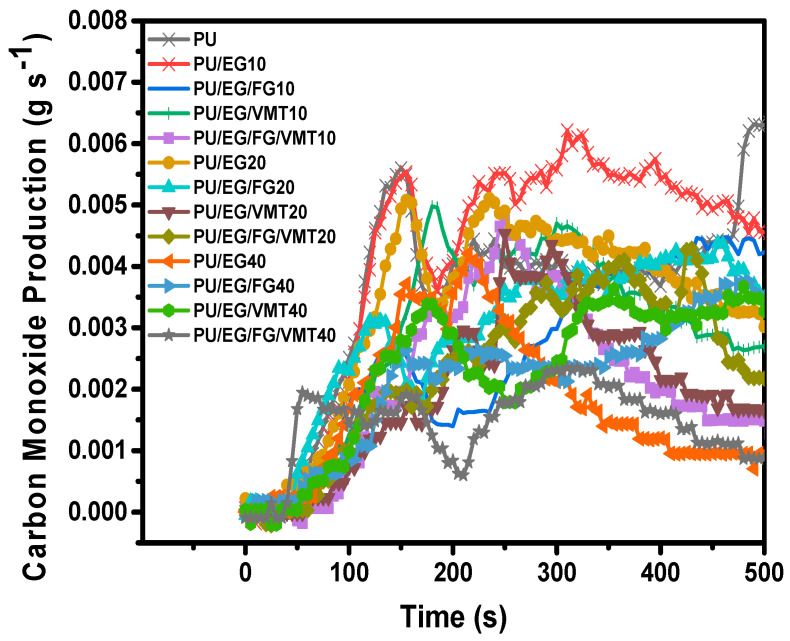
CO as a function of the burning time for PU composite samples.

Carbon monoxide (CO) is a deadly gas that heavily contributes to the mortality observed in fires, and its production should be limited in enclosed spaces. In Figure 8, the values vary for all the samples and a trend is difficult to observe. The PU/EG/FG/VMT40 sample presents the lowest peak, 0.025 gs^−1^. However, the other 40 wt.% samples exhibit higher peaks, showing that the combination of all the additives may produce lower levels of CO. The EG and VMT act together with their charring properties to prevent thermal degradation. Most of the samples peak at between 0.04 gs^−1^ and 0.05 gs^−1^, with the PU/EG10 and PU samples peaking at 0.06 gs^−1^. Most of the samples fall from their peaks but continue to sustain CO production after 500 s.

### 2.5. Char Residue

**Figure 9:**
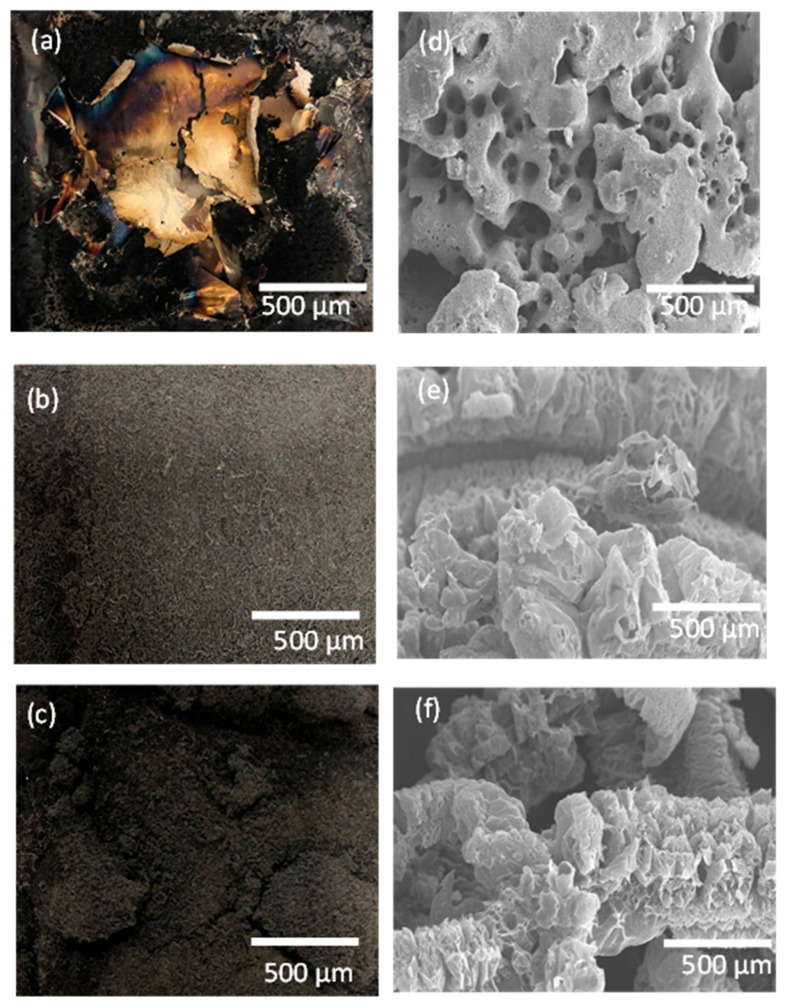
Photographs of char formed after cone calorimeter test for the control (**a**) PU, (**b**) PU/EG10, and (**c**) PU/EG/FG/VMT40; SEM images (25 k) of the char residues after cone calorimeter test for the control (**d**) PU, (**e**) PU/EG10, and (**f**) PU/EG/FG/VMT40.

To display the effect of char formation on the combustion behaviour of the FR-PU composite materials, the morphology and the structure of the chars after the CCT were captured using a digital camera and SEM. Figure 9a–c show digital photographs of the residual char from the control PU, PU/EG10, and PU/EG/FG/VMT40 samples. The raw PU, PU/EG10, and PU/EG/FG/VMT40 samples were selected to demonstrate how the hybrid chars of the three instrumental FR chemicals formed compared with the control matrix. Figure 9a shows that the control PU has no charring layer and most of the material has burnt away, which is expected owing to the porous nature of flexible PU allowing for fast combustion [24,27]. It can be observed that “worm-like” intumescent and dense char was left after the addition of EG (PU/EG10 and PU/EG/FG/VMT40). The EG samples have a thicker, fluffier “worm-like” char, which provides a good barrier from the heat of the cone apparatus [18]. Figure 9b shows the PU/EG10 sample, which has a uniform char, while the PU/EG/FG/VMT40 (Figure 9c) has a thick char, larger ripples, and cracking. This is due to the EG particles not being as homogenously distributed within the reinforcement materials (VMT and FG) at the higher concentration. These variations in surface morphology allow combustion fuel and heat to penetrate the layer and burn the PU below [28]. This morphed shape of the sample and air movement from the cone apparatus meant that a great deal of the char disintegrated, allowing for more combustion. This over-expansion and blow-off is known as the popcorn effect and is a known consequence of high weightings of additives in PU [24,25,26].

Figure 9d–f show the SEM images of the char residue for the control PU, PU/EG10, and PU/EG/FG/VMT40. The SEM image of PU (Figure 9d) appears to have a thicker surface morphology than the PU composites owing to the thermo-oxidation degradation of polymer chains during the combustion process. This is clearly observed in the image of the char residue in Figure 9a, where the material has almost completely burnt away. The surface morphology of Figure 9e PU/EG10 shows a larger, more intact charring structure of the sample, which reflects its strong intumescent effect. Homogeneity and compactness of the char are good indicators of effective intumescence in SEM imagery [29], which correlate with Figure 9b. The relationship between these larger sections of char and effective flame retardant behaviour is also observed in other SEM investigations using EG and APP [29]. Figure 9f demonstrates that EG, VMT, and FG are incorporated in PU/EG systems. The main hypothesis for this is that EG, VMT, and FG can decompose to form silica, a self-extinguishing layer, when exposed to heat, and, as the materials decompose, it leaves inorganic silica residue. These silica layers are non-volatile viscous semisolid materials that can cover the matrix surface to form a liquid layer and can catalyse the dehydration carbonisation of the PU matrix [30]. The formed viscous liquid layer can reinforce the densification and strength of the “worm-like” intumescent char layer (Figure 9c,f) and enhance adhesion with the PU matrix at the same time; hence, they are good indicators of effective intumescence FR materials, as supported by the CCT data. The PU/EG/FG/VMT40 sample was shown to char (Figure 9c) and prevent a high pHRR (Figure 4) but continued to smoulder, generating excess smoke and heat up to 450 s later than the PU/EG10 and control PU.

## 3. Experimental Section

### 3.1. Materials

Table 2 provides details of the materials for the experimental procedure. A two-part (A/B) polyurethane elastomer (PU-WC565) with a Shore-A hardness of 65A (65A SPU) was provided by Barnes Pt. Australia. Fillers were incorporated in part A (composition mixtures: cycloaliphatic polymer, methylene bis(4-cyclohexylisocyanate), naphtha petroleum, light aromatic solvent, xylene; 1,2,4-trimethyl benzene, ethylbenzene, *N*-methyl-2-pyrrolidone, toluene, phenylmercuric acetate) of the PU and homogeneously mixed using a mechanical blade mixer operating at 300 rpm for 5 min. Part B (composition mixtures: polyether polyol mixture, *N*-methyl-2-pyrrolidone, phenylmercuric acetate) of the PU was then added to the above mixture to cross-link the composite and mixed for a further 5 min. The mixture was degassed and poured into a mould. To cure these composites, moulds were kept in a laboratory at room temperature for 24 h. In this work, 12 samples were fabricated and subjected to combustibility and mechanical measurements. Detailed formulation of each sample is provided in Table 3.

### 3.2. Sample Characterisation

Scanning electron microscopy (SEM) (FEI NOVA NANO 230; Boston, USA) was conducted to investigate the dispersion of the fillers, the morphology of the cross-section area, and the char residue (after cone calorimetry testing (CCT)) of these samples, with an accelerating voltage of 5 kV. The transverse sections and the char residue (after the cone calorimetry test) were coated with a thin platinum layer (20 nm in thickness).

The tensile tests were performed with a strength testing machine (Instron 34 T-30kN; Boston, MA, USA). A static load cell of 1 kN was used for tensile testing with a crosshead velocity of 50 mm/min. Tensile testing samples were fabricated into a dog bone shape according to ASTM D412-C standard. For each group, at least three samples were fabricated. For each tensile experiment, the load and displacement were recorded. The experiments were conducted at room temperature (23 °C).

Thermogravimetric analysis (TGA) was performed using a Perkin Elmer Simultaneous Thermal Analyzer (STA 6000; Boston, MA, USA) with a temperature range from 30 °C to 800 °C and a heating rate of 10 °C min^−1^ under air atmosphere.

Cone calorimeter measurements were performed using an Atlas Cone 2 (iCone Classic (Fire Testing Technology; London, UK) according to ASTM E 1354 at incident flux of 35 kW/m^2^ using a cone-shaped heater. The spark was continuous until the sample ignited. All samples (100 × 100 × 25 mm) were exposed to a heat flux of 35 kW/m^2^.

### 3.3. Combustion Mechanism

**Scheme 1:**
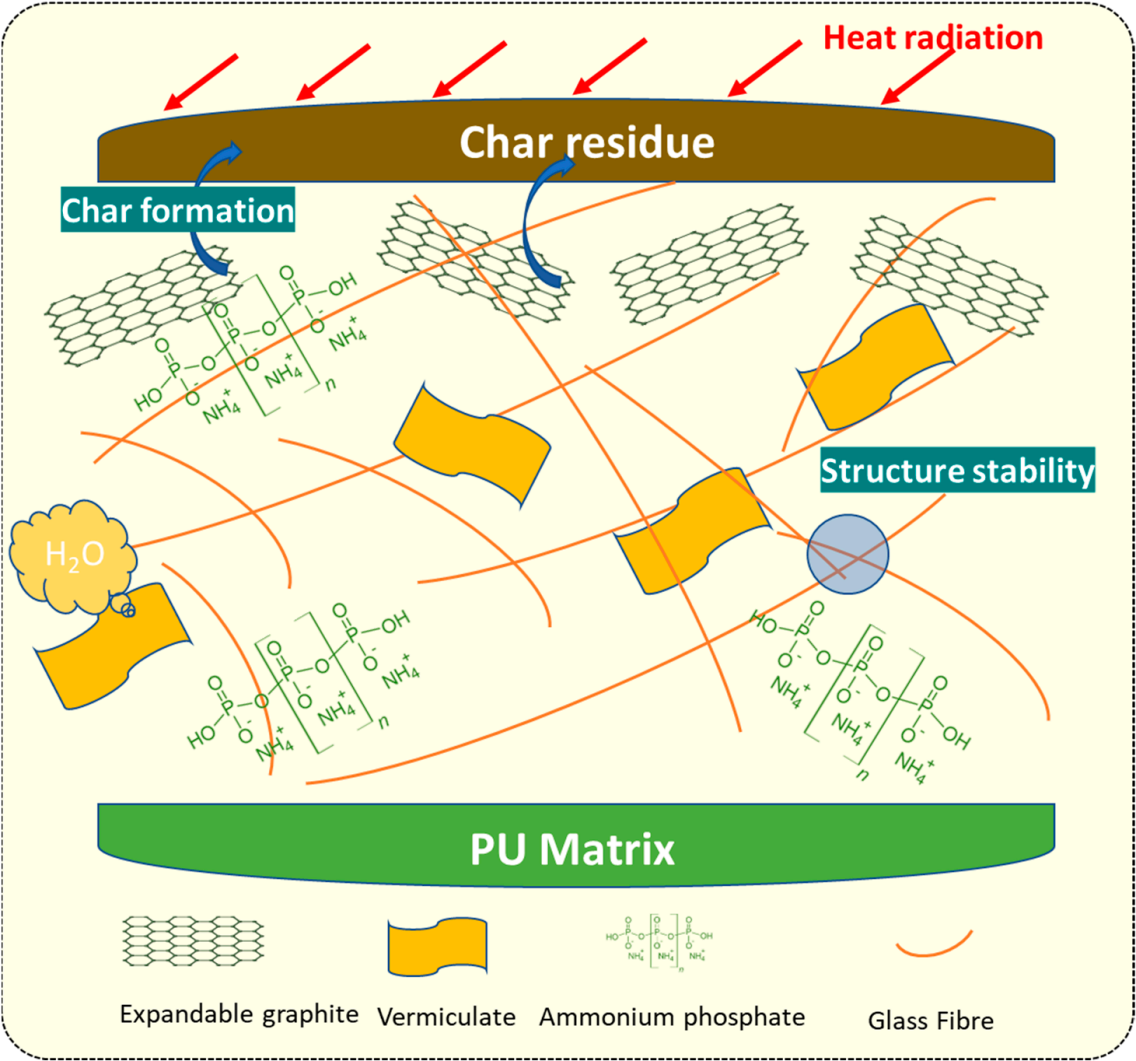
Synthesis of PU composites’ mechanism.

Scheme 1 shows the synthesis of PU composite mechanisms used in this study, where each component provides a different functionality. The PU polymer matrix (part A and B) is thermoplastic and flexible. EG, which consists of a layered structure, with sulfuric acid (H_2_SO_4_) intercalated between the graphite flakes, oxidises the H_2_SO_4_ upon heating to expand the carbon layer into a thick char. This char functions as a smoke suppressor. APP comprises phosphorus, which alters the thermal degradation process to carbonise and form char layers through the condensable phase reaction. The FG structure has enough protection ability to shield it from alkali agents and was reinforced in the combustion process to maintain the mechanical strength of the polymer composites while increasing their IFR properties. VMT consists of a hydrous silicate material (triclinic structure), which, upon heating (>200 °C), will exfoliate and release H_2_O between the mineral layers and produce a further char protective layer. This is the primary flame retardant mechanism of the PU composite of this study.

## 4. Conclusions

In this work, the flame retardant properties of a new PU composite containing EG, APP, VMT, and FG were investigated for use in fire-resistant applications for the first time. Through TGA analysis under an oxygen gas environment, it was found that the PU composites with 40 wt.% EG had greater thermal stability than those with 20 w.t.% and 10 w.t.% EG along with APP, VMT, and FG in the PU composite. Tensile strength testing showed that the control PU withstood the most stress compared with the PU composite samples. The samples with 40 wt.% EG had the highest yield point owing to fibreglass reinforcement with EG in the PU polymer. Flammability analysis and flame retardancy properties of all PU composites were assessed using cone calorimetry testing. The PU composites proved far superior compared with the control PU tested over a wide range of flammability test parameters. The cone calorimetry test showed that the values of pHRR, SPR, TSR, CO_2_, and CO were reduced after the incorporation of 40 wt.% EG along with APP, VMT, and FG and showed compact and close char layers, which functioned as a barrier to retard the combustion process. The char acted as an insulating and non-burning material, reducing the emission of volatile products into the flame area. Overall, the results showed that the addition of 40 wt.% EG along with APP, VMT, and FG delayed the combustion process, which was attributed to the excellent dispersion and barrier effect of EG in PU. This work demonstrates that using these additives in combination in PU composites has the potential to be developed to produce cost-effective, low-toxicity, and environmentally benign materials for building elements to impart flame resistance performance.

For future work, X-ray photoelectron spectroscopy (XPS) may be utilized to detect the elemental composition with an instrument able to penetrate samples within more than 2–5 nm, and the UL-94 test can be completed to more completely characterize the fire resistance of the studied materials [31]. As the introduction of fillers may affect the viscosity of the composition [13], assessment of the viscosity is recommended in future studies to determine the properties of the new materials. Furthermore, VMT and FG evidenced the capability of improving the FR and heat insulation of the composite. Further studies need to be conducted to assess the suitable concentrations.

## Figures and Tables

**Table 1:** Tensile results for PU composites and control PU.

Sample	Average Elongation (mm)	Young’s Modulus (MPa)	Ultimate Tensile Strength (MPa)
Pure PU	78.96 ± 0.35	2.86 ± 0.25	2.96 ± 0.27
PU/EG10	52.14 ± 0.27	5.33 ± 0.02	1.80 ± 0.17
PU/EG40	7.14 ± 0.15	15.05 ± 0.03	1.35 ± 0.18
PU/EG/FG10	54.65 ± 0.22	5.69 ± 0.02	2.21 ± 0.15
PU/EG/FG40	12.79 ± 0.12	17.57 ± 0.06	1.81 ± 0.12
PU/EG/VMT10	53.04 ± 0.15	5.63 ± 0.05	1.77 ± 0.09
PU/EG/VMT40	5.11 ± 0.06	34.78 ± 0.12	1.91 ± 0.04
PU/VMT/FG/EG10	32.73 ± 0.21	9.25 ± 0.03	1.75 ± 0.07
PU/VMT/FG/EG40	8.23 ± 0.02	29.17 ± 0.04	1.73 ± 0.02

**Table 2:** Details of the materials used for developing the PU composites’ formulations.

Polyurethane Elastomer (PU-WC565)	Part (A/B), Shore-A Hardness: 65 A	Barnes Pty. AU
Ammonium polyphosphate (APP)	AP 422 (phase II)- Powder (≤100 µm)	Clariant
Expandable Graphite (EG)	Carbonfoil-FF 80-Flakes (≤250 µm)	Metachem Manuf Co.
VMT	Powder (≤100 µm)	Allnex Compos (AU)
FG	Flakes (≤450 µm)	Allnex Compos (AU)

**Table 3:** Formulation of PU composites.

Sample Code	PU (Mass%)	EG (Mass%)	FG (Mass%)	VMT (Mass%)
Pure PU	100	--	--	--
PU/EG10 *	70	10	--	--
PU/EG/FG10 *	65	10	5	--
PU/EG/VMT10 *	65	10	--	5
PU/EG/FG/VMT10 *	60	10	5	5
PU/EG20 *	60	20	--	--
PU/EG/FG20 *	55	20	5	--
PU/EG/VMT20 *	55	20	--	5
PU/EG/FG/VMT20 *	50	20	5	5
PU/EG40 *	40	40	--	--
PU/EG/FG40 *	35	40	5	
PU/EG/VMT40 *	35	40	--	5
PU/EG/FG/VMT40 *	30	40	5	5

* APP remains constant at 20% (mass) for all PU composites’ formulations.

## Data Availability

No new data were created.
